# Nonuniform Video Size Reduction for Moving Objects

**DOI:** 10.1155/2014/832871

**Published:** 2014-08-31

**Authors:** Anh Vu Le, Seung-Won Jung, Chee Sun Won

**Affiliations:** ^1^Department of Electrical and Electronic Engineering, Dongguk University-Seoul, Seoul 100-715, Republic of Korea; ^2^Department of Multimedia Engineering, Dongguk University-Seoul, Seoul 100-715, Republic of Korea

## Abstract

Moving objects of interest (MOOIs) in surveillance videos are detected and encapsulated by bounding boxes. Since moving objects are defined by temporal activities through the consecutive video frames, it is necessary to examine a group of frames (GoF) to detect the moving objects. To do that, the traces of moving objects in the GoF are quantified by forming a spatiotemporal gradient map (STGM) through the GoF. Each pixel value in the STGM corresponds to the maximum temporal gradient of the spatial gradients at the same pixel location for all frames in the GoF. Therefore, the STGM highlights boundaries of the MOOI in the GoF and the optimal bounding box encapsulating the MOOI can be determined as the local areas with the peak average STGM energy. Once an MOOI and its bounding box are identified, the inside and outside of it can be treated differently for object-aware size reduction. Our optimal encapsulation method for the MOOI in the surveillance videos makes it possible to recognize the moving objects even after the low bitrate video compressions.

## 1. Introduction

Surveillance cameras are ubiquitous and play an important role in our daily life. The recorded video data from the surveillance cameras provide rich information to many applications ranging from human and machine interactions [1–3] to content indexing and retrieval [4, 5]. For such applications of digital video surveillance and digital video recording (DVR) systems [6, 7], it is often required to examine moving objects for a long period of frames in recorded videos. This naturally demands highly efficient compressions for a huge amount of video data. Here, the conflicting requirement is how to maintain high visual quality, especially for the important information in the video such as the moving objects, in low bit-rate compressions.

A wide range of advanced techniques has been proposed to improve the conventional video compression framework. For example, an efficient block mode determination algorithm [8] was applied for an efficient scalable video compression, where video data can change their resolution to use the limited bandwidth efficiently. The scalable compression scheme is particularly useful for surveillance videos. Note that surveillance videos usually consist of alternating sequence of frames with static background and moving objects. Definitely, the moving objects are the important data to be preserved in the compressions. This requires the compression technique to distinguish the important moving objects of interest (MOOI) from the unimportant static background (non-MOOI) in the video and to treat them differently in the compression process. As a result, the natural user interface (NUI) via the face detection [9, 10] in the surveillance videos can be a feasible technique even for highly compressed videos. To differentiate the MOOI from the non-MOOI, the object segmentation and tracking processes can be applied [6, 7]. However, these methods need to identify accurate object boundaries, which often require expensive computations. Weng et al. [11] used Kalman filter for object detecting and tracking. This method can detect and track the object trajectory frame by frame accurately. However, the object boundary that differentiates the MOOI from the non-MOOI cannot be identified clearly. Goswami et al. [12] used a mesh-based technique to track moving objects in video sequence. Mesh-based motion estimation techniques are more accurate than the block based method, but they are relatively slow due to the high computational complexity.

**Figure 1:**
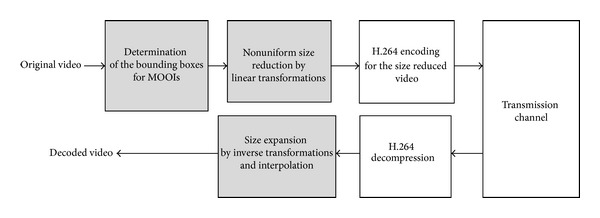
Flowchart of the proposed video encoding and decoding system.

In this paper we differentiate the MOOI from the non-MOOI by detecting the bounding boxes surrounding the MOOIs for each group of frames (GoF). Then, the detected bounding boxes encapsulating the MOOIs are fixed throughout the GoF. To detect the bounding box we need to identify the pixels with spatiotemporal saliency. For this, we construct the spatiotemporal gradient map (STGM) of a GoF [13], where each pixel in the STGM represents the level of the temporal and spatial saliency. Then, the optimal size of the bounding box is determined to include the local pixels with the highest energy density of the STGM. Once the pixels including the MOOI are determined by the bounding boxes, we can apply linear transformations with different slopes to the inside and outside of the bounding boxes such that the MOOI is intact while those in the non-MOOI are the main target for size reduction. After this initial data reduction, the standard H.264/AVC compression is applied to the size-reduced frames for further compressions. At the receiver, the reverse processes including decompression by H.264/AVC and the size expansion by the inverse linear transformations are applied to restore the video data with the original size. The overall block diagram of our MOOI-based compression is shown in Figure 1.

As far as the image size reduction is concerned, various methods have been proposed for content-aware image and video retargeting context such as the seam carving methods [13–17]. These methods reduce the image size by removing the unimportant seam lines that have the low saliency. The output video has the reduced spatial resolution, where the rich texture areas are maintained but the homogenous areas are removed. These video retargeting methods are mainly for display purposes and it is not reversible to reconstruct the original image size from the retargeted videos unless the decoder knows exact locations of the discarded seam pixels. Therefore, the conventional video retargeting methods are not appropriate as the initial data reduction for video compression. Note that image pruning scheme with image downsampling as a preprocessing step of video compressions has been also used in Vo et al. [18], where one of the two consecutive image lines (i.e., even or odd lines) is to be discarded for image size reduction. Since the line dropping is limited for one of two consecutive lines and the criterion for line dropping is based on the least mean square errors (LMSE) of the interpolated image data, it is hard to differentiate the MOOI from the non-MOOI. A reversible nonuniform size reduction method was also proposed in Won and Shirani [19] without the bounding box. Finally, we note that this paper is the extended version of our previous single MOOI [20] to multiple MOOIs.

Our contributions of this paper can be summarized as follows: (i) we introduce a spatiotemporal gradient map (STGM) to trace the boundary of the MOOI within a GoF; (ii) based on the STGM, a cost function for determining the center and size of the bounding box encapsulating the MOOI is formulated; (iii) an optimization process for updating the center and size of the bounding box alternately is introduced; (iv) the subjective visual quality especially for the MOOI is enhanced by nonuniformly reducing the size of the video frames as a preprocessing for the H.264 video compressions.

This paper is organized as follows. In Section 2, the algorithm for detecting multiple moving objects in video is presented. Then, different linear transforms are applied to MOOI and non-MOOI for size reduction in Section 3.  Section 4 shows the experimental results of proposed method.  Section 5 concludes this paper.

## 2. Detection of Multiple Moving Objects of Interest

Moving objects in video can be detected by motion estimation, which is a computationally expensive process. Instead, in this paper, all MOOIs are detected by using spatial and temporal gradients in a GoF of H.264/AVC structure. Specifically, a spatial gradient map *S*_*g*_^*t*^(*i*, *j*) at pixel (*i*, *j*) of a frame *I* ^*t*^ with a size of *N*_*r*_ × *N*_*c*_ can be defined as an average of the magnitude of spatial gradients within a window (2*ψ* + 1)×(2*ψ* + 1) as follows:

(1) $$\begin{array}{c} S_{g}^{t}\left(i,j\right)=\frac{1}{{\left(2\psi +1\right)}^{2}}\overset{\psi }{\underset{u=-\psi }{\sum }}\overset{\psi }{\underset{v=-\psi }{\sum }}g^{t}\left(i+u,j+v\right), \end{array}$$

where *g* ^*t*^ = |∂*I* ^*t*^/∂*i*| + |∂*I* ^*t*^/∂*j*| is the magnitude of the spatial gradient. Using *S*_*g*_^*t*^, we define the temporal saliency cost *S*_tsc_^*t*^ by computing the temporal gradient of the spatial gradients between the two consecutive even numbered frames (even numbered frames give more temporal deviations with less computational load):

(2) $$\begin{array}{c} S_{\text{tsc}}^{t}=\left|S_{g}^{t}-S_{g}^{t+2}\right|. \end{array}$$

As a result of the spatiotemporal gradients in (1) and (2), the boundary pixels of the moving objects are highlighted in the temporal saliency cost *S*_tsc_^*t*^. Then, we can construct a STGM *R* ^*k*^ by mosaicking the maximum saliency cost *S*_tsc_^*t*^ at each pixel location throughout the GoF with even numbered frames starting from the first even numbered frame *m*_0_ to *m*_0_ + *N*_0_ − 2 for *N*_0_ frames in the* k*th GoF of the H.264/AVC as follows:

(3) $$\begin{array}{c} R^{k}\left(i,j\right)=S_{\text{tsc}}^{t^{*}}\left(i,j\right),\; \end{array}$$

where t* = arg max⁡_*t*∈[*m*_0_,*m*_0_+*N*_0_−2]_⁡*S*_tsc_^*t*^(*i*, *j*). Note that, according to the definition of *R* ^*k*^(*i*, *j*) in (3), the value of *R* ^*k*^(*i*, *j*) corresponds to the spatial or temporal boundary of the MOOI in the GoF. Therefore, we can define a bounding box (or window) with (2*w*_*r*_ + 1)×(2*w*_*c*_ + 1) to encompass the trace of each MOOI, where the weighted sum of *R* ^*k*^(*i*, *j*) within the optimal window at the center *C* = (*C*_*r*_, *C*_*c*_) yields the peak value. Therefore, our problem boils down to the determination of the optimal window size (*w*_*r*_, *w*_*c*_) and the center of the bounding box *C* = (*C*_*r*_, *C*_*c*_). In this paper, we propose a novel method to find bounding boxes and their centers for multiple MOOIs in a GoF. Our approach to determine (*w*_*r*_, *w*_*c*_) and *C* = (*C*_*r*_, *C*_*c*_) is to take an alternate optimization process between (4) and (5). That is, starting from an initial value of (*w*_*r*_^(0)^, *w*_*c*_^(0)^) we apply (4) to have (*C*_*r*_^(1)^, *C*_*c*_^(1)^). Then (*C*_*r*_^(1)^, *C*_*c*_^(1)^) is used to update (*w*_*r*_^(1)^, *w*_*c*_^(1)^) by (5). This alternate process continues until there is no more change from (*w*_*r*_^(*n*−1)^, *w*_*c*_^(*n*−1)^) to (*w*_*r*_^(*n*)^, *w*_*c*_^(*n*)^). Consider

(4) (Cr(n),Cc(n)) =arg max⁡1≤i≤Nr,1≤j≤Nc{∑u=−wr(n−1)wr(n−1)∑v=−wc(n−1)wc(n−1)Rk(i+u,j+v)},

(5) $$\begin{array}{c} \left(w_{r}^{\left(n\right)},w_{c}^{\left(n\right)}\right)=\underset{\begin{array}{c} w_{\min}\leq w_{r}\leq w_{\max}\\ w_{\min}\leq w_{c}\leq w_{\max} \end{array}}{\arg\;\max}\left\{B_{w_{r},w_{c}}^{\text{edge}}\chi \left(B_{w_{r},w_{c}}^{\text{non-edge}}<T_{l}\right)\right\}, \end{array}$$

where

(6) $$\begin{array}{c} B_{w_{r},w_{c}}^{\text{edge}}=\overset{w_{r}}{\underset{u=-w_{r}}{\sum }}\overset{w_{c}}{\underset{v=-w_{c}}{\sum }}\chi \left(R^{k}\left(C_{r}^{\left(n\right)}+u,C_{c}^{\left(n\right)}+v\right)>T_{g}\right),\\ \;\;\;\;B_{w_{r},w_{c}}^{\text{non-edge}}=\overset{w_{r}}{\underset{u=-w_{r}}{\sum }}\overset{w_{c}}{\underset{v=-w_{c}}{\sum }}\chi \left(R^{k}\left(C_{r}^{\left(n\right)}+u,C_{c}^{\left(n\right)}+v\right)<T_{g}\right) \end{array}$$

and *χ* is an indicator function such that

(7) $$\begin{array}{c} \chi \left(\varphi \right)=\{\begin{array}{cc} 1, & \text{if}\;\varphi \;\text{is}\;\text{True}\\ 0, & \text{otherwise}. \end{array} \end{array}$$

**Figure 2:**
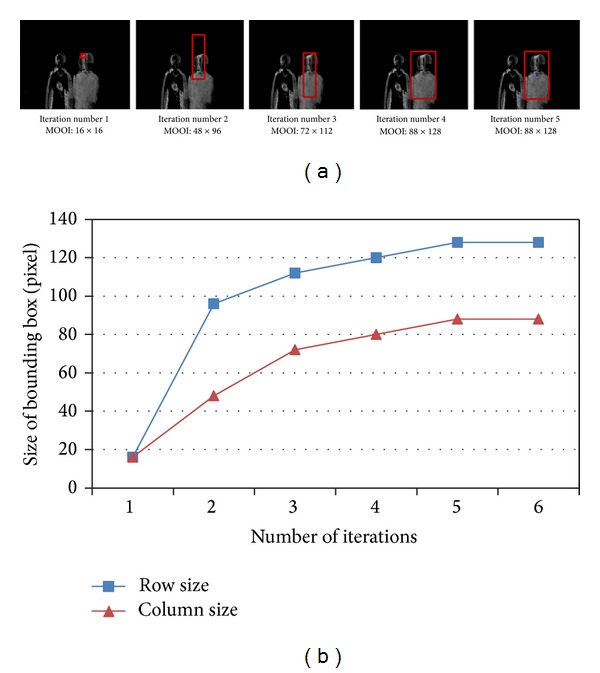
Convergence of the alternating optimization process: (a) detected bounding boxes for different iteration numbers and (b) convergence of the alternating optimization method with respect to the size and the number of iterations.

*T*_*g*_ and *T*_*l*_ are predetermined thresholds. Note that, given the current window size (*w*_*r*_^(*n*−1)^, *w*_*c*_^(*n*−1)^), we find the center of the bounding box (*C*_*r*_^(*n*)^, *C*_*c*_^(*n*)^) for all pixels in the image by (4). Then, given the current center (*C*_*r*_^(*n*)^, *C*_*c*_^(*n*)^) of the bounding box, we examine all possible window sizes to find the maximum number of strong edges within the window (i.e., *B*_*w*^*r*^,*w*^*c*^_^edge^ in (5)) under the condition that the number of weak edges is less than a threshold *T*_*l*_ (i.e., *B*_*w*^*r*^,*w*^*c*^_^non-edge^ in (5)).  Figure 2 shows the convergence of our alternate optimization process of (4) and (5) for *w*_min⁡_ = 16 and *w*_max⁡_ = 128, where our method converges after about the 5th iteration. Note that the center and the size of the bounding box improve after every iterative step. This tells that the center and size of the bounding box are updated cooperatively, which eventually leads to the final convergence. Because our method is based on a binary decision by the threshold, the computation for each iteration is very simple and the convergence is very fast.

For the case of multiple MOOIs in the GoF, after the first MOOI (denoted as MOOI-1) and its bounding box are defined, the bounding box for the next MOOI (i.e., MOOI-2) can be found by repeating the alternating optimization process of (4) and (5). This time, in order not to detect the already-found bounding box again we set the pixel values of the STGM under the predetermined bounding boxes to zeroes before we search for the next MOOI (i.e., MOOI-2). This search process for the next MOOIs and their optimal bounding boxes continues until the sum of all pixel values of the STGM under the bounding box is less than a threshold *T*_*s*_. After all moving objects and their bounding boxes in the GOF are found, we have *P* multiple MOOIs from MOOI-1 to MOOI-*P*.

## 3. Linear Transformations for Nonuniform Size Reduction

After all MOOIs and their bounding boxes in each GOF are determined, we can reduce the sizes for MOOIs and non-MOOIs nonuniformly. In this paper, to treat the inside and outside regions of the bounding boxes differently and to speed up the size reduction process, linear transformations with different slopes for MOOIs and non-MOOIs are applied. So, to squeeze the original frame of *N*_*r*_ × *N*_*c*_ to *M*_*r*_ × *M*_*c*_(*M*_*r*_ < *N*_*r*_ and *M*_*c*_ < *N*_*c*_) we first apply 1D linear transformations to reduce the number of rows from *N*_*r*_ to *M*_*r*_. Subsequently, the number of columns is reduced from *N*_*c*_ to *M*_*c*_ to have the squeezed frame of *M*_*r*_ × *M*_*c*_. Since these sequential 1D reductions for the rows and columns are similar, we describe only the row reduction in this section.

**Figure 3:**
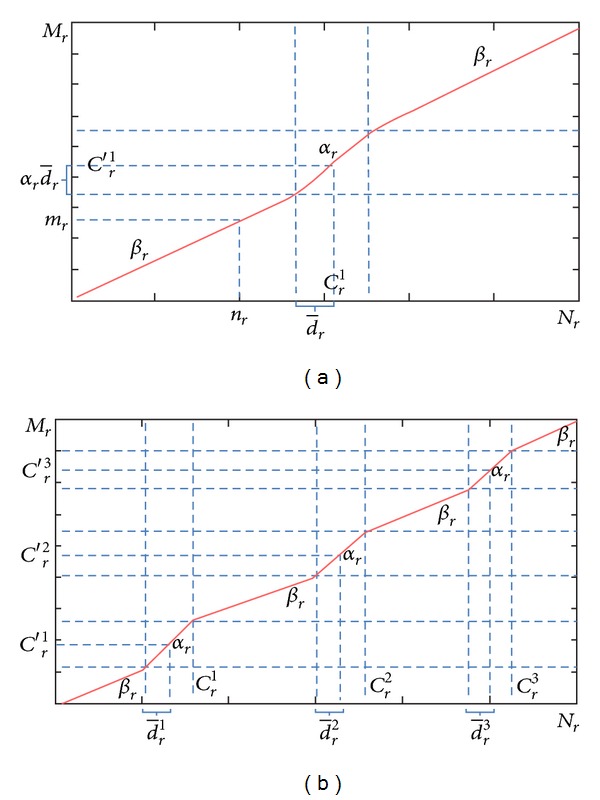
Linear transformation for (a) single MOOI and (b) three MOOIs.

For each row *n*_*r*_  (1 ≤ *n*_*r*_ ≤ *N*_*r*_) we need a linear mapping function *S*_row_[*n*_*r*_] to convert the original row index *n*_*r*_ to *S*_row_[*n*_*r*_]  (1 ≤ *S*_row_[*n*_*r*_] ≤ *M*_*r*_). Depending on the existence of the MOOI at *n*_*r*_ or not, the slope of the linear function *S*_row_[*n*_*r*_] takes either *α*_*r*_ for the MOOI or *β*_*r*_ for the non-MOOI (see Figure 3). So, the slopes control the amount of the size reduction between the MOOI and the non-MOOI and we have *β*_*r*_ < *α*_*r*_ ≤ 1. Specifically, for the *p*th MOOI (i.e., MOOI-*p*), 1 ≤ *p* ≤ *P*, we denote *d* ^*p*^(*n*_*r*_) = |*C*_*r*_^*p*^ − *n*_*r*_| as the absolute distance from the center of MOOI-*p*, *C*_*r*_^*p*^, to the row index *n*_*r*_ in the original frame and ${\overset{-}{d}}_{r}^{p}$ represents one-half of the vertical size of the MOOI-*p*. Also, *C*_*r*_^′*p*^ denotes the row index of the center of the MOOI-*p* at the reduced frame. Note that the index *p* of the MOOI-*p* is assigned sequentially from the left to the right of the image space and we start the linear mapping with MOOI-1 by the following linear transformation *S*_row_[*n*_*r*_] for each row *n*_*r*_ in $1\leq n_{r}<C_{r}^{1}+{\overset{-}{d}}_{r}^{1}$:

(8) Srow[nr]={Cr′1−αrd−r1−βr(d1(nr)−d−r1)if  0<nr<Cr1−d−r1,Cr′1−αrd1(nr)if  Cr1−d−r1≤nr<Cr1,Cr′1+αrd1(nr)if  Cr1≤nr<Cr1+d−r1.

Then, for the next rows in $C_{r}^{p}+{\overset{-}{d}}_{r}^{p}\leq n_{r}\leq C_{r}^{p+1}-{\overset{-}{d}}_{r}^{p+1}$ with *p* ≥ 1 we have the following mapping function *S*_row_[*n*_*r*_]:

(9) Srow[nr] ={Cr′p−αrd−rp+Cr′p−1+αrd−rp−1+βr(dp(nr)−d−rp)  if  Crp−1+d−rp−1≤nr<Crp−d−rp,Cr′p−αrdp(nr)  if  Crp−d−rp≤nr<Crp,Cr′p+αrdp(nr)  if  Crp≤nr<Crp+d−rp,Cr′p+αrd−rp+βr(dp(nr)−d−rp)  if  Crp+d−rp≤nr<Crp+1−d−rp+1.

Finally, for the rows in the last MOOI-*P* $C_{r}^{P}-{\overset{-}{d}}_{r}^{P}\leq n_{r}\leq N-1$ we have the mapping function *S*_row_[*n*_*r*_] as follows:

(10) $$\begin{array}{c} S_{\text{row}}\left[n_{r}\right]=\{\begin{array}{cc} C_{r}^{\prime P}-\alpha_{r}d^{P}\left(n_{r}\right) & \\ \;\;\;\text{if}\;C_{r}^{P}-{\overset{-}{d}}_{r}^{P}\leq n_{r}<C_{r}^{P}, & \\ C_{r}^{\prime P}+\alpha_{r}d^{P}\left(n_{r}\right) & \\ \;\;\;\text{if}\;C_{r}^{P}\leq n_{r}<C_{r}^{P}+{\overset{-}{d}}_{r}^{P}, & \\ C_{r}^{\prime P}+\alpha_{r}{\overset{-}{d}}_{r}^{P}+\beta_{r}\left(d^{P}\left(n_{r}\right)-{\overset{-}{d}}_{r}^{P}\right) & \\ \;\;\;\text{if}\;C_{r}^{P}+{\overset{-}{d}}_{r}^{P}\leq n_{r}\leq N_{r}-1. & \end{array} \end{array}$$

Given the reduction rate *α*_*r*_ for the MOOIs, the reduction rate for the non-MOOI *β*_*r*_ should be determined by considering the overall reduction ratio from *N*_*r*_ to *M*_*r*_ as well as *α*_*r*_. So, for a single MOOI, *β*_*r*_ is given by

(11) $$\begin{array}{c} \beta_{r}=\frac{M_{r}-1-2{\overset{-}{d}}_{r}^{1}\alpha_{r}}{N_{r}-1-2{\overset{-}{d}}_{r}^{1}}. \end{array}$$

For multiple MOOIs *β*_*r*_ is calculated as follows:

(12) βr=Mr−1−2d−r1αr−∑l=2p2d−rlαrCr1−d−r1+Nr−1−Crp−d−rp+∑l=2pCrl−d−rl−Crl−1−d−rl−1.

The index of the center of the MOOI is also changed from *C*_*r*_^*p*^ to *C*_*r*_^′*p*^ after the reduction. Specifically, the indices for the first MOOI and the next MOOIs are given as the following equations, respectively:

(13) Cr′1 =βr(Cr1−d−r1)+αrd−r1,

(14)  Cr′p=Cr′p−1+αrd−r1+βr(Crp−d−rp−Crp−1−d−rp−1)+αrd−rp.

**Figure 4:**
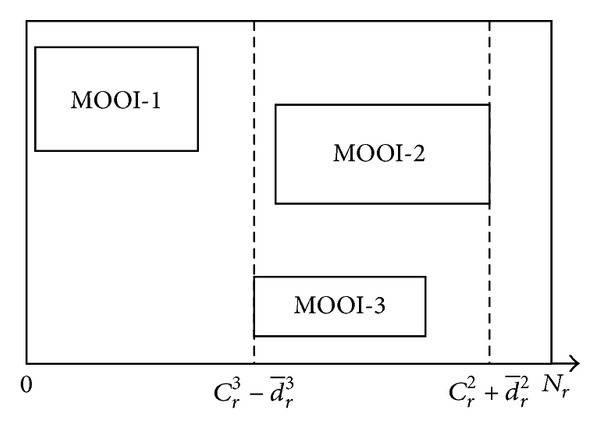
Illustration of the case when the two bounding boxes are overlapped horizontally.

In practice, bounding boxes of MOOIs can be overlapped horizontally and/or vertically. Specifically, we define that bounding boxes are overlapped when their 1D projections are overlapped. This is because 1D linear transformations are applied to rows and columns separately.  Figure 4 shows the example when the bounding boxes are overlapped horizontally. To deal with the overlapped bounding boxes, the boundaries of MOOI-2 and MOOI-3 are merged; that is, new left and right boundaries and center are determined as $C_{r}^{3}-{\bar{d}}_{r}^{3}$, $C_{r}^{2}+{\bar{d}}_{r}^{2}$, and $\left(C_{r}^{2}+C_{r}^{3}+{\bar{d}}_{r}^{2}-{\bar{d}}_{r}^{3}\right)/2$, respectively. The row reduction is then performed using (8)–(14) for MOOI-1 and the merged MOOI. The column reduction is performed in a similar manner.

Our goal of the linear transformations is to keep the original image data in the MOOIs as much as possible after the size reduction, while achieving the major size reduction in the non-MOOI. Therefore, we first set *α*_*r*_ ≈ 1 and adjust *β*_*r*_ (*β*_*r*_ < *α*_*r*_) to meet the requirement of the size reduction. After the transformations of (8)–(10) the integer valued indices at the reduced rows are determined by the interpolation from the actual mapped indices. After the row reduction, the transformation-interpolation process is applied to the columns to complete the size reduction.

After the size reduction, the conventional H.264/AVC is used to further compress the size-reduced frames and the compressed bit stream is sent to the receiver. At the receiver, after decoding compressed bit-stream, the decompressed frames are expanded to the original size by the inverse transformation-interpolation for the columns and the rows sequentially. The sizes of bounding boxes, their centers, and the size reduction rate of MOOIs are sent to the decoder as side information for the size expansion. Note that we can use the same GoF boundaries as those from the H.264/AVC.

## 4. Experimental Results

Our experiments have been conducted to demonstrate two aspects: (i) accuracy of the proposed MOOI detection with the bounding box and (ii) usefulness of the proposed MOOI detection. The accuracy of the proposed MOOI detection with the bounding box is judged by visual comparisons with the previous inter-frame based Kalman filtering approach [11]. The usefulness of the proposed MOOI detections is demonstrated by applying the detected MOOI to the content-aware image resizing with the comparison of the LMSE method [18] and to the image size reduction as a preprocessing for the H.264/AVC compressions.

The surveillance video sequences [21, 22] were used to evaluate the performance of the proposed method. In all our experiments, the parameters are predetermined and fixed as follows: *ψ* = 3, *w*_min⁡_ = 16,  *w*_max⁡_ = 128, *T*_*l*_ = 0.8(2*w*_max⁡_ + 1)(2*w*_max⁡_ + 1), *T*_*g*_ = 0.09, *T*_*s*_ = (2*w*_min⁡_ + 1)(2*w*_min⁡_ + 1)/2. The threshold parameters affect the accuracy of the bounding box detection. The users can interact with the system by adjusting these parameter values. For comparisons, the proposed method and the LMSE method in Vo et al. [18] were applied to reduce the size of the video frames before we apply H.264/AVC. Then, the visual qualities after the H.264/AVC decompression and the size expansion are compared.

**Figure 5:**
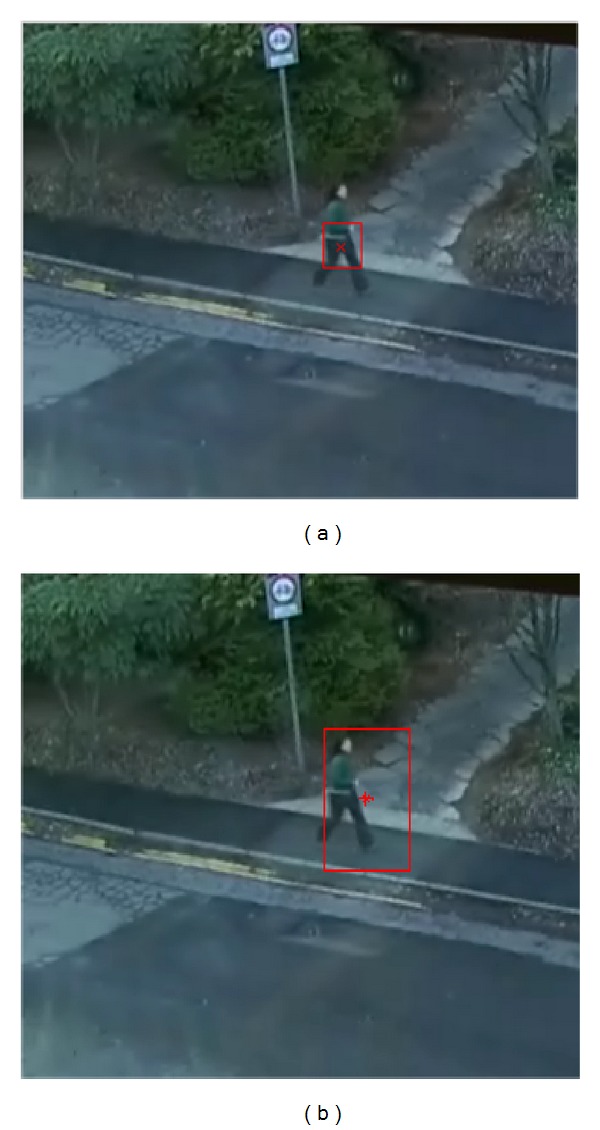
Comparison for the detection of boundary box: (a) Kalman filtering object detection and tracking [11] (b) the proposed method.

**Figure 6:**
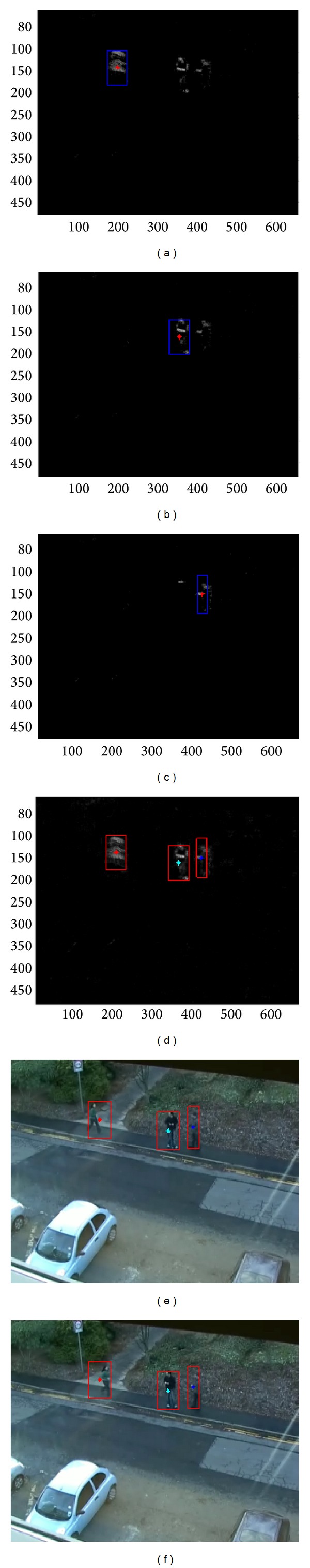
Proposed bounding box detection for multiple moving objects in a GOF: (a) first MOOI (MOOI-1), (b) second MOOI (MOOI-2), (c) third MOOI (MOOI-3), (d) bounding boxes for all MOOIs in the STGM, (e) bounding boxes for the first frame of the GoF, and (f) bounding boxes for the last frame of the GoF.

The proposed bounding box detection method is also compared to the moving object detecting and tracking method [11]. As shown in Figure 5, the Kalman filtering method [11] tends to detect only the moving part between the consecutive frames not the whole body of the moving object, which demonstrates the power of our STGM-based formulation of the cost function for a GoF. For the case of multiple MOOIs, Figure 6 shows the order of MOOI detection from the first MOOI (i.e., MOOI-1 in Figure 6(a)) at the leftmost side of the image to the last MOOI (i.e., MOOI-3 in Figure 6(c)) at the rightmost side of the image in a GoF. This demonstrates the extension of our previous work [20] to the problem of multiple MOOIs. Since our bounding box is determined on the basis of the GoF, the first bounding box of the walking person includes all pixels along the motion trajectories from the first frame (Figure 6(e)) to the last one (Figure 6(f)) of the GoF.

**Figure 7:**
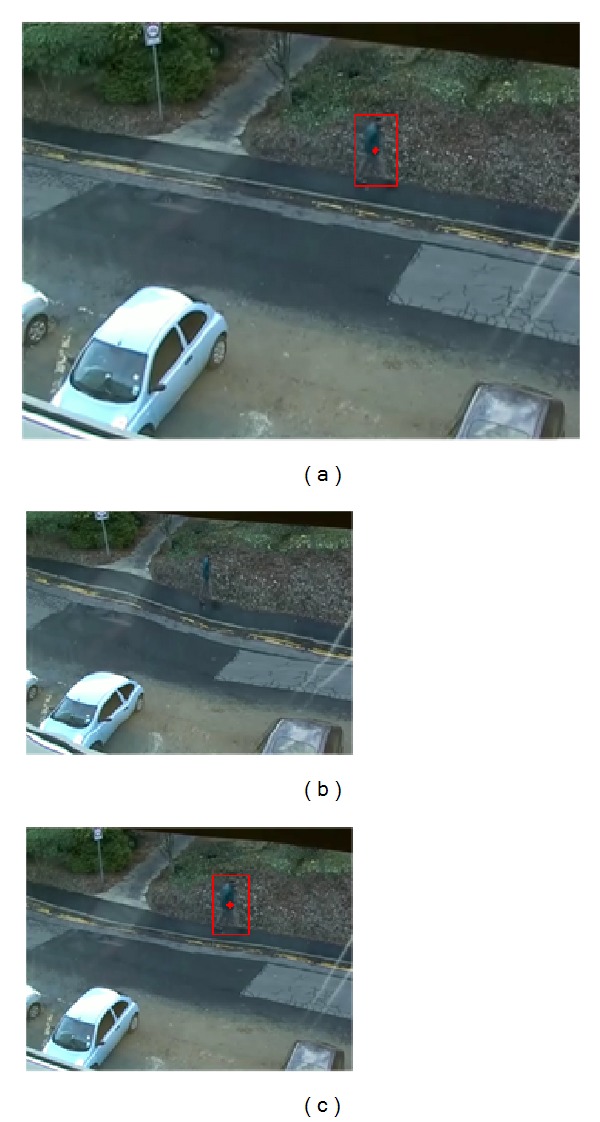
Size reduction by 30% with a single MOOI: (a) original, (b) LMSE [18], and (c) the proposed method.

**Figure 8:**
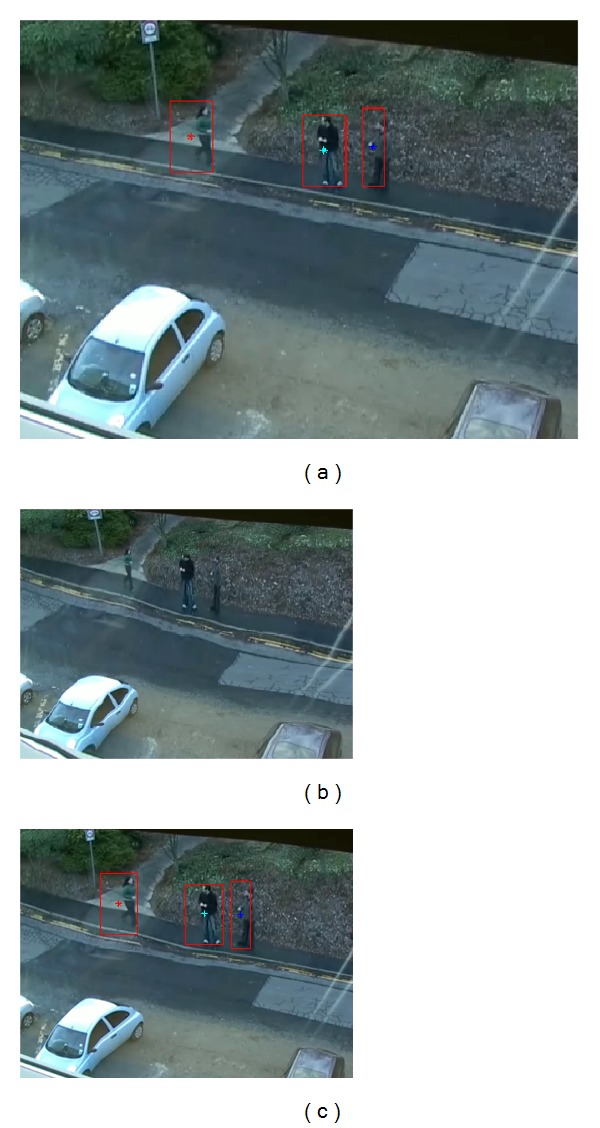
Size reduction by 30% with three MOOIs: (a) original, (b) reduced frame by LMSE [18], and (c) the proposed method.

**Figure 9:**
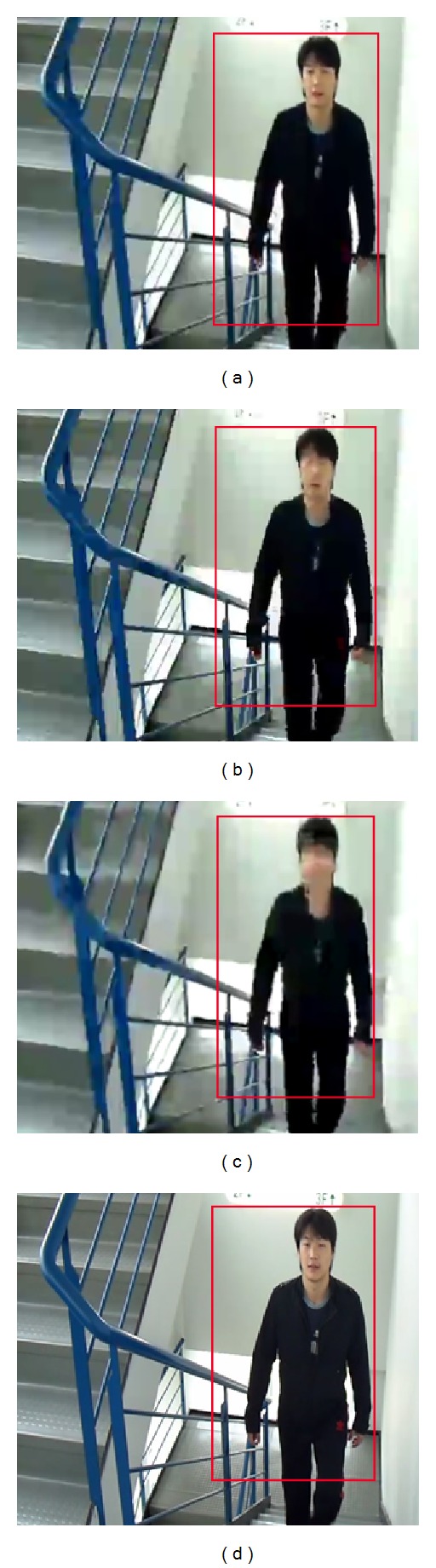
300th frame of Stair sequence: size reduction by 30% and bitrate 100 kbps (MOOI inside the bounding box): (a) original, (b) H.264/AVC without the size reduction, (c) the LMSE [18], and (d) the proposed method.

**Figure 10:**
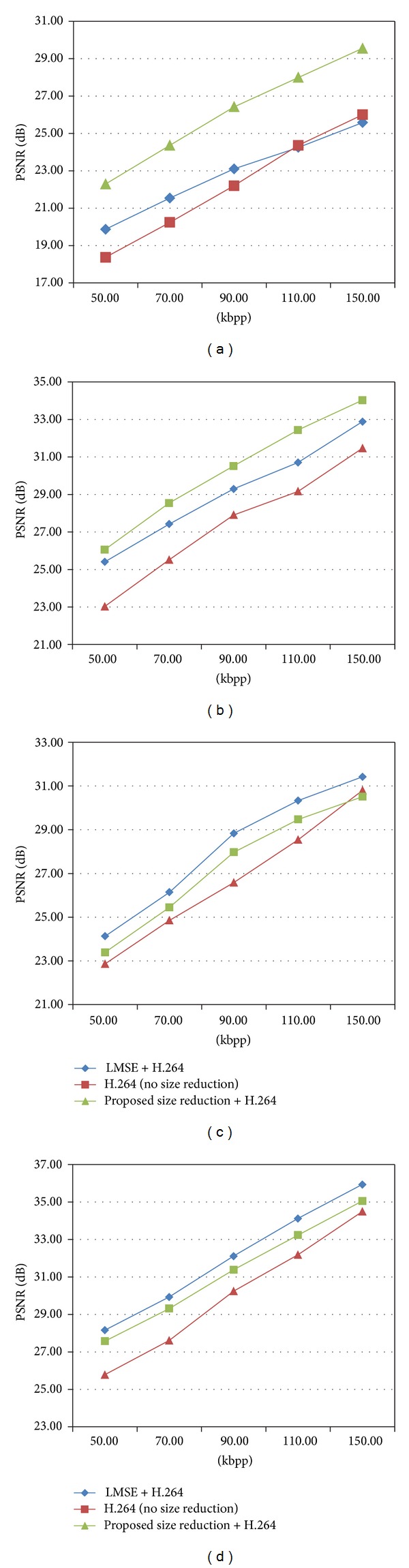
Rate and distortion graphs: (a) Stair sequence for the MOOI only, (b) Hallway sequence for the MOOI only, (c) Stair sequence for the whole image, and (d) Hallway for the whole image.

Once the MOOIs are detected with the bounding boxes, we can differentiate the image regions of the moving objects from the rest of the image regions of nonmoving objects. This allows us to treat MOOIs and non-MOOI separately for image size reduction. That is, we can nonuniformly reduce the size of the image frames in the video before compressions such that the non-MOOIs are the major target for the size reduction. Frame reductions by 30% and bitrates of 50–200 kbps were tested for the visual comparisons of the MOOIs after the decompressions and size expansions.  Figure 7 and Figure 8 show the results of the size reduction by the LMSE in Vo et al. [18] and our method for a single MOOI and three MOOIs, respectively. As shown in the figures, the moving objects are almost intact after the size reduction by the proposed method.  Figure 9 for the Stair sequence in the database [21] demonstrates the differences more clearly. As one can see, the proposed method outperforms the LMSE inside regions of the MOOI in terms of PSNR and visual quality.  Figure 10 compares the numerical results by the rate-distortion graphs. Although our method yields PSNR slightly lower than the LMSE for the whole image, inside the MOOI regions, it achieves 3~4 dB higher PSNRs than the LMSE and the H.264/AVC compressions without the size-reduction at bitrates lower than critical bitrate of 150 kbps.

## 5. Conclusions

Optimal bounding box detection method for the moving object of interest (MOOI) has been proposed. Multiple MOOIs as well as a single one can be automatically detected by the proposed method. Once the bounding boxes are identified, one can treat the MOOI and the non-MOOI differently to preserve the visual quality of the important MOOIs. Linear transformations with different slopes are used to nonuniformly reduce the sizes of the MOOIs and non-MOOI. Our size reduction method can be applied as an initial compression for the H.264/AVC video compression standard. Experimental results show that the decompressed videos of the H.264/AVC using the proposed method yield better PSNRs for the MOOI about 3 dB higher than the LMSE.
